# *The Botucatu Document*: One health antifungal resistance policies — A call for action

**DOI:** 10.1016/j.onehlt.2026.101319

**Published:** 2026-01-06

**Authors:** Paulo Cezar Ceresini, Waldir Cintra de Jesus Junior, Ana Carolina Firmino, Bárbara Pereira Christofaro Silva, Danilo Tancler Stipp, Edson Luiz Furtado, Fábio Luiz Checchio Mingotte, Karine Assis Costa, Paulo Renato Matos Lopes, Rita Luiza Peruquetti

**Affiliations:** a Universidade Estadual Paulista (UNESP), Faculdade de Engenharia, Câmpus de Ilha Solteira, Ilha Solteira, SP, Brazil; b Universidade Federal de São Carlos (UFSCar), Centro de Ciências da Natureza, Câmpus Lagoa do Sino, Buri, SP, Brazil; c Universidade Estadual Paulista (UNESP), Faculdade de Ciências Agrárias e Tecnológicas, Câmpus de Dracena, Dracena, SP, Brazil; d Universidade Estadual Paulista (UNESP), Faculdade de Ciências Agrárias e Veterinárias, Câmpus de Jaboticabal, Jaboticabal, SP, Brazil; e Universidade Estadual Paulista (UNESP), Faculdade de Ciências Agronômicas, Câmpus de Botucatu, Botucatu, SP, Brazil

**Keywords:** Antifungal resistance, *Aspergillus fumigatus*, One health, Agricultural fungicides, Antimicrobial resistance governance, Environmental surveillance, Clinical diagnostics, Fungicide regulation, Public health policy, AMR surveillance

## Abstract

Antifungal resistance in *Aspergillus fumigatus* and other environmental fungi represents a growing global threat to human health, driven in part by agricultural fungicide use. The scale of the threat is masked by inadequate multisector surveillance. In December 2025, the Brazilian Network Meeting on *Aspergillus fumigatus* Antimicrobial Resistance convened clinical, agricultural, environmental, and public-health experts to address these challenges using a One Health framework. Through a structured plenary deliberation, participants approved “*The Botucatu Document*: One Health Antifungal Resistance Policies — A Call for Action,” a unified Public Statement of Concern outlining governance principles and coordinated national actions for Brazil. The Statement reflects consensus across four Working Groups on clinical surveillance, environmental monitoring, fungicide regulation, and public health communication. Together, these directives call for independent AMR data collection, FAIR transparency, cross-sector governance, strengthened laboratory capacity, environmental aerobiome surveillance, new fungicide risk-assessment criteria, and comprehensive One Health communication strategies. This manuscript presents the full Public Statement as approved verbatim, situates it within global AMR policy frameworks, and highlights implications for Brazil's forthcoming National AMR Action Plan (2026–2031). The *Botucatu Document* represents a milestone in aligning agricultural and clinical sectors around a shared One Health AMR agenda.

## 1 Introduction

Fungal diseases are an underrecognized yet increasingly significant contributor to global morbidity and mortality. Recent global analyses estimate that severe fungal diseases cause substantial mortality annually, surpassing many bacterial infections in scale [1]. Among them, *Aspergillus fumigatus* is of particular concern due to its ubiquity, its capacity to cause life-threatening infections, and its increasing resistance to frontline triazoles used in clinical practice [2,3]. Environmental selection—particularly through the widespread use of agricultural azoles—has been implicated in the emergence of triazole-resistant *A. fumigatus*, with agricultural and clinical resistance mechanisms often converging [[4], [5], [6]]. International reports have also documented clonal expansion of multi-fungicide-resistant *A. fumigatus*, illustrating the ecological connectivity between environmental and clinical resistance evolution [7].

Brazil, one of the world's largest users of agricultural fungicides, has reported increasing detection of clinical azole-resistant *A. fumigatus*, including well-known mutations such as TR₃₄/L98H and novel *cyp51A* variants [5,[8], [9], [10], [11], [12]]. Studies from Latin America further reinforce the growing regional burden of azole resistance [10]. At the same time, Brazil lacks an integrated national system for environmental and clinical surveillance, coordinated AMR data governance, or harmonized policies linking agricultural fungicide regulation to public health needs. This gap persists despite strong global guidance emphasizing One Health integration [[13], [14], [15]] and growing evidence of environmental origins of clinical resistance [2,4,5,16].

Brazil has formally recognized antimicrobial resistance (AMR), including fungal AMR, as a major public-health and agricultural challenge under a One Health framework. Through the *National Action Plan for the Prevention and Control of Antimicrobial Resistance in the Agricultural Sector* (PAN-BR AGRO), the Ministry of Agriculture and Livestock (MAPA) acknowledges that AMR threatens human, animal, and environmental health, and that national policies must align with quadripartite guidance from the World Health Organization (WHO), the Food and Agriculture Organization of the United Nations (FAO), the World Organization for Animal Health (WOAH), and the United Nations Environment Programme (UNEP) [17]. The first quinquennium of PAN-BR AGRO (2018–2022) established foundational actions in surveillance and stewardship, while its second stage emphasizes the need for stronger cross-sectoral integration, transparency, and improved risk governance. Despite these commitments, fungal AMR—particularly azole-resistant *Aspergillus fumigatus*—remains insufficiently addressed in agricultural and environmental policy.

In response, a national expert meeting on *Aspergillus fumigatus* antimicrobial resistance, held in Brazil in December 2025, brought together specialists across human health, plant pathology, microbiology, epidemiology, public policy, and environmental sciences. The meeting was designed to accelerate the development of One Health strategies to prevent antifungal resistance, inform regulatory action, and strengthen Brazil's forthcoming National AMR Action Plan (PAN-BR 2026–2031). Four Working Groups addressed: (1) clinical surveillance and diagnostics, (2) environmental AMR monitoring, (3) fungicide regulatory frameworks, and (4) One Health public communication strategies.

The result is *The Botucatu Document*, a unified Public Statement of Concern approved by the plenary and presented here verbatim.

## 2 Methods: consensus-building process

The Meeting followed a structured consensus methodology to ensure transparency, inclusiveness, and scientific rigor.

### 2.1 Meeting preparation

Participants received the meeting proceedings containing the briefing materials summarizing the global evidence on antifungal resistance, existing Brazilian surveillance systems, agricultural fungicide use, and One Health policy frameworks (WHO–FAO–WOAH). Experts were invited both from Brazil and abroad, representing clinical mycology, plant pathology, molecular biology, epidemiology, public health, agricultural research, and regulatory science.

### 2.2 Working group deliberations

Participants were assigned to four Working Groups (WG-A–D), each with a specific mandate:

WG-A: Clinical surveillance and diagnostic capacity.

WG-B: Environmental surveillance for *A. fumigatus.*

WG-C: Fungicide regulatory strategies.

WG-D: One Health communication and advocacy.

Each group generated a written recommendation as a single “core suggestion” summarizing its essential contribution.

### 2.3 Plenary approval procedure

Plenary participants were all accredited national and international experts and institutional members formally invited to contribute scientifically or technically to this Meeting. It was not part of the voting body: industry representatives, media, logistical staff, and non-accredited observers. A formal, stepwise plenary approval process was followed:

**Step 1 — Approval of One Health governance principles.**

Governance standards were approved first, establishing the foundation for all subsequent decisions.

**Step 2 — Approval of the structure of the Statement.**

The plenary approved the Statement's architecture, consisting of the four WG sections and integrated recommendations.

**Step 3 — Approval of the core suggestions from WGs A–D.**

Each core suggestion was reviewed, discussed, refined where needed, and approved by vote.

**Step 4 — Approval of the Public Statement of Concern as a whole.**

The final unified document (*The Botucatu Document*) was adopted as the official output of the Meeting. No signatures were required; endorsement reflects the plenary approval process itself.

### 2.4 Protection of the integrity of the public statement

To maintain fidelity to the democratic and scientific process used in the Meeting, the Public Statement included in this opinion paper is presented verbatim, with no alterations or reinterpretations.

## 3 Public statement of concern (approved verbatim)

“Antifungal resistance is a growing global threat affecting human health, agriculture, and environmental integrity. Recognizing Brazil's pivotal role as a major agricultural producer and a country facing increasing clinical incidence of antifungal-resistant *Aspergillus*, the participants of the Brazilian Network Meeting on *Aspergillus fumigatus* AMR present this Public Statement of Concern. This statement calls for strengthened One Health governance, transparent surveillance, responsible regulation, and coordinated communication strategies to protect public health while ensuring agricultural sustainability.

This Statement is not a legal or institutional commitment. It represents a collective summary of the discussions and concerns raised during the meeting, offered for consideration by national authorities and the broader One Health community.

### 3.1 One Health governance principles

#### 3.1.1 Independence of data collection

National AMR surveillance must rely primarily on independently collected data. Industry-generated datasets may be useful as supplementary information but cannot replace independent surveillance systems intended to guide public health policy.

#### 3.1.2 FAIR public transparency

All surveillance outputs should be made publicly available following FAIR principles: Findable, Accessible, Interoperable, and Reusable.

#### 3.1.3 Standardized and auditable methods

Sampling strategies, laboratory workflows, and analytical methods should follow harmonized, documented, quality-assured, and auditable protocols to ensure reproducibility and national comparability.

#### 3.1.4 Public funding and public oversight

Surveillance infrastructure and analytical capacity must be publicly governed and sustained by public resources. Where private funding exists, oversight and decision-making must remain exclusively public and multisectoral.

#### 3.1.5 One Health integration

Human, animal, agricultural, and environmental sectors must be jointly engaged in surveillance, regulation, and communication to ensure comprehensive risk management.

Alignment With PAN—BR 2026–2031: All strategies should reinforce and support Brazil's commitments under the upcoming national AMR action plan.

### 3.2 Core suggestions from the working groups

#### 3.2.1 WG-A – Clinical surveillance & public health measures

Brazil urgently needs to strengthen national capacity for diagnosing aspergillosis and detecting antifungal resistance through standardized diagnostic methods, national reporting, public data accessibility, and integration of resistance rates into One Health regulatory frameworks.

#### 3.2.2 WG-B – Environmental surveillance

Brazil should establish an independent, national environmental surveillance system beginning with a pilot program based on standardized aerobiome methodologies. Surveillance must be conducted by publicly funded, independently operated laboratories. Industry-generated datasets may support but cannot substitute national surveillance.

#### 3.2.3 WG-C – One Health regulation of agricultural fungicides

The regulatory system must incorporate One Health criteria so that fungicides with known or plausible cross-resistance risks to clinical antifungals undergo strengthened risk assessment and precautionary regulatory measures.

#### 3.2.4 WG-D – Communication strategy

A strong One Health communication strategy is essential. Brazil should align messaging with PAN—BR 2026–2031, engage communication professionals, partner with AMR networks, and implement audience-specific educational strategies for schools, universities, farmers, and the general public.

### 3.3 Public Statement Of Concern

This document reflects the collective deliberations of the plenary and summarizes the key summary recommendations of the event. It does not imply institutional endorsement by any participant or organization and contains no signature requirement.”

## 4 Discussion

*The Botucatu Document* represents a landmark in Brazil's efforts to integrate agricultural, clinical, and environmental sectors into a unified One Health antifungal resistance strategy. Its directives are consistent with global AMR warnings that environmental selection pressures—particularly agricultural triazole use—contribute to clinically significant resistance [[4], [5], [6],^16^].

The emphasis on independent AMR data collection responds to well-established concerns about the limitations of privately generated datasets for public-health decision making [4,6,16,18]. The Document's emphasis on FAIR transparency, exclusively public and multisectoral oversight—encompassing the human, animal, agricultural, and environmental health sectors—and harmonized methodologies is strongly aligned with WHO–FAO–WOAH guidance for fungal AMR governance [[13], [14], [15]].

WG-A's recommendation for expanding clinical diagnostic capacity and adopting standardized antifungal susceptibility testing builds on long-recognized diagnostic challenges in invasive aspergillosis [21], while responding to contemporary evidence that persistent underdiagnosis is associated with resistance-related mortality [12,19,20].

WG-B's environmental surveillance recommendation places Brazil at the frontier of aerobiome-based AMR detection, an approach increasingly recognized for its ability to detect environmental evolution in fungal pathogens [3,6,7].

WG-C's recommendations bring Brazil closer to risk-based regulatory frameworks advocated internationally, especially where agricultural fungicides may exert selective pressure on human-pathogenic fungi [[4], [5], [6],^16^].

WG-D highlights that technical progress is insufficient without public awareness and political momentum—an insight reinforced globally in AMR policy analyses [13,22].

Together, these components position Brazil to contribute meaningfully to global antifungal resistance mitigation and to strengthen its National AMR Action Plan (PAN-BR 2026–2031). They demonstrate that antifungal resistance is not purely a medical or agricultural issue but a systemic governance challenge requiring coordinated, transparent, whole-of-society action.

## 5 Conclusion

*The Botucatu Document* embodies a historic consensus among Brazilian and international experts regarding the urgent need to reform surveillance, regulation, and communication around antifungal resistance. By integrating agricultural fungicide risk assessment with clinical and environmental AMR surveillance, Brazil has taken a major step toward aligning national policy with global One Health standards.

The Document provides an actionable framework for the 2026–2031 National AMR Action Plan and sets a precedent for multisector coordination in fungal AMR governance. Continued investment, interdisciplinary collaboration, and political commitment will be essential to transform these recommendations into measurable public-health protection.

## Meeting proceedings

Full program information, speaker contributions, and Working Groups are publicly available at https://www.feis.unesp.br/#!/aspergillus. The meeting proceedings were formally archived as a printed supplement of the journal Summa Phytopathologica (SP), the official publication of the *Associação Paulista de Fitopatologia* (São Paulo State Plant Pathology Association).

## The origin of the name

The Public Statement of Concern resulting from this process was designated *The Botucatu Document* to reflect its origin in Botucatu, São Paulo State, Brazil, where the plenary deliberations and formal approval took place. The designation follows the tradition of consensus documents named after their place of adoption, emphasizing collective authorship and geographic context rather than individual attribution.

## CRediT authorship contribution statement

**Paulo Cezar Ceresini:** Writing – review & editing, Writing – original draft, Supervision, Resources, Project administration, Methodology, Funding acquisition, Conceptualization. **Waldir Cintrade Jesus Junior:** Writing – review & editing, Supervision, Project administration, Methodology, Conceptualization. **Ana Carolina Firmino:** Writing – review & editing, Resources, Project administration, Methodology. **Bárbara Pereira Christofaro Silva:** Writing – review & editing, Resources, Project administration, Methodology. **Danilo Tancler Stipp:** Writing – review & editing, Resources, Project administration, Methodology. **Edson Luiz Furtado:** Writing – review & editing, Resources, Project administration, Methodology. **Fábio Luiz Checchio Mingotte:** Writing – review & editing, Resources, Project administration, Methodology. **Karine Assis Costa:** Writing – review & editing, Resources, Project administration, Methodology. **Paulo Renato Matos Lopes:** Writing – review & editing, Resources, Project administration, Methodology. **Rita Luiza Peruquetti:** Writing – review & editing, Resources, Project administration, Methodology.

## Ethics statement

Ethical review and approval were not required for this study as it does not involve human participants, animals, or identifiable personal data.

## Funding

The development of this opnion paper was supported by resources from the Brazilian Network Meeting on *Aspergillus fumigatus* Antimicrobial Resistance, held at UNESP Botucatu Campus (3–5 December 2025), and by institutional support from 10.13039/501100009568Universidade Estadual Paulista (UNESP) Pro-Rectory of Extension and Culture (PROEC) and Universidade Federal de São Carlos (UFSCar). This meeting and preparatory work were supported by the Fungal AMR Innovations for Low and Middle-Income Countries (LMICs) – FAILSAFE program (UKRI/10.13039/501100000265Medical Research Council – Centre for Medical Mycology), 10.13039/501100002322Coordination for the Improvement of Higher Education Personnel – CAPES, and the Brazilian National Research Council - CNPq (310316/2021-9, 307193/2022-5**,** and 311895**/**2022-0), /2022-0), Brazil. The funders had no involvement in the deliberations, conclusions, or drafting of the Public Statement of Concern. The publication fee will be covered through internal research funds allocated for scientific dissemination.

## Declaration of competing interest

The authors declare no commercial or financial relationships that could be construed as a potential conflict of interest.

## Data Availability

No new datasets were generated or analyzed for this study.
